# Do Gynecologists-Obstetricians Know How to Manage Pregnancies in Women After Bariatric Surgery?

**DOI:** 10.3390/healthcare13101107

**Published:** 2025-05-09

**Authors:** Joanna Dzedzej, Maria Szewczyk, Jan Pietruszka, Mateusz Ficygowski, Agnieszka Majewska, Wojciech Migal, Jan Dębski, Maciej Walędziak, Anna Różańska-Walędziak

**Affiliations:** 1 Faculty of Medicine, Collegium Medicum, Cardinal Stefan Wyszynski University in Warsaw, 01-938 Warsaw, Poland; j.dzedzej@student.uksw.edu.pl (J.D.); mariaszewczyk97@gmail.com (M.S.); janpietruszka112@gmail.com (J.P.); m.ficygowski@student.uksw.edu.pl (M.F.); a.majewska@student.uksw.edu.pl (A.M.); w.migal@student.uksw.edu.pl (W.M.); jdebski@student.uksw.edu.pl (J.D.); 2 Department of General, Oncologic, Metabolic, and Thoracic Surgery, Military Institute of Medicine, Szaserów 128, 04-141 Warsaw, Poland; 3 Department of Human Physiology and Pathophysiology, Faculty of Medicine, Collegium Medicum, Cardinal Stefan Wyszynski University in Warsaw, 01-938 Warsaw, Poland; aniaroza@tlen.pl

**Keywords:** bariatric surgery, gestational diabetes mellitus, vitamin and micronutrient deficiencies, diet in pregnancy, pregnancy

## Abstract

**Introduction and aim of the study**: The majority of bariatric procedures are performed in women of reproductive age, which has resulted in a growing number of pregnancies following bariatric surgery. The purpose of the study was to analyze whether obstetricians-gynecologists taking care of pregnancies after bariatric surgery possess sufficient knowledge about it. **Materials and method**: The study was conducted in the form of a multiple choice, anonymous survey, conducted among 123 certified specialists in obstetrics and gynecology and 37 residents training in obstetrics and gynecology. The questions were related to supplementation of vitamins and micronutrients, the appropriate time interval between bariatric surgery and pregnancy, dietary recommendations, diagnosing gestational diabetes mellitus, and risks for the fetus following bariatric surgery. **Results**: The majority of participants did not know the correct answers, with only one participant who answered correctly all the questions provided. There was a correlation between giving correct answers to the question about dietary recommendations and the question about risks existing during a pregnancy after bariatric surgery. Only 30% of participants knew how gestational diabetes mellitus should be diagnosed in pregnancies after bariatric surgery. **Conclusions**: To enhance safety for women and their children, it is of utmost importance to implement additional training and educational programs for obstetricians-gynecologists about optimum care for pregnancies after bariatric surgery.

## 1. Introduction

Recent studies from 2022 conducted by the World Health Organization (WHO) suggest that 2.5 billion people are overweight, and that 890 million adults are living with obesity, indicating that one in eight people are living with obesity. Obesity is typically identified using the Body Mass Index (BMI), defined as a BMI ≥ 30 kg/m^2^.

The rising prevalence of obesity can be attributed to multiple factors, including unhealthy lifestyle, lack of physical activity, poor dietary habits, and genetic as well as environmental factors. Obesity has numerous adverse health consequences, significantly impacting both the quality and length of life of affected individuals. Increased body weight is strongly associated with a higher risk of developing chronic conditions such as type 2 diabetes, hypertension, cardiovascular diseases, and different types of cancer, including breast, colorectal, and prostate cancer. Additionally, obesity is a key driver in the development of metabolic syndrome, a cluster of conditions characterized by dysregulated lipid and carbohydrate metabolism and cardiovascular complications, ultimately contributing to increased mortality rates [1]. Obesity constitutes a significant health risk during pregnancy, and the prevalence of obese pregnant women in Poland between 2012 and 2017 exhibited an upward trend, increasing from 5.5% to 7.5% [2].

Bariatric surgery (BS) is a recommended method of treatment for obesity, offering sustained weight loss, symptom reduction, and improvements in obesity-related co-morbidities. Bariatric treatment is indicated when other treatment modalities, such as dietary interventions, lifestyle changes, or pharmacotherapy, have proven ineffective. Bariatric surgery facilitates weight loss in numerous ways, by reducing gastric volume, decreasing ghrelin secretion and influencing intestinal absorption of nutrients, leading to weight loss and reduced fat tissue volume, and significant metabolic and hormonal changes. It is estimated that approximately 80% of bariatric procedures are performed in women of reproductive age, which has resulted in a growing number of pregnancies following BS [3]. Elevated BMI during pregnancy is associated with adverse outcomes for both the mother and baby, including higher risk of fetal macrosomia, gestational diabetes mellitus, pregnancy-induced hypertension, and fetal malformations, as well as higher risk of labor complications [4]. Women affected with obesity require professional support and care to minimize the risk of complications during and after pregnancy.

BS, while effective in reducing body weight and leading to remission of co-morbidities, often results in nutritional deficiencies, including inadequate intake of vitamins and micronutrients, necessitating a specialized diet during pregnancy [5,6]. The inability to perform an Oral Glucose Tolerance Test (OGTT) requires alternative diagnostic approaches for gestational diabetes mellitus (GDM) [7]. OGTT is not recommended to be performed after bariatric surgery as it may result in hypoglycemia, which is especially harmful in pregnant women, as it may lead to fetal death. Additionally, the results of OGTT are not credible after BS due to absorption and intestinal motility changes. Given the increasing population of post-bariatric women, questions arise regarding gynecologists’ and obstetricians’ knowledge of nutritional requirements, caloric needs, food portion size, the timing of solid food and liquid intake, and the consumption of high-protein meals. Improper management of such pregnancies may lead to micronutrient deficiencies, which may ultimately contribute to fetal growth restriction (FGR) [8].

The purpose of the study was to find an answer to the following question: Do Polish gynecologists-obstetricians possess sufficient expertise to manage pregnancies after BS effectively, despite the limited guidelines provided by the International Federation for the Surgery of Obesity and Metabolic Disorders (IFSO) and Polish recommendations? A secondary purpose of the study was to assess whether there were any differences in the level of knowledge between specialists in obstetrics and gynecology and residents who were still in training.

## 2. Materials and Methods

The study was conducted in the form of an anonymous survey, available both online and in a paper version, aimed at optimizing care for pregnant patients following bariatric surgery. The survey was distributed in October 2023, during the international 17th Postgraduate Academy Congress for specialists in obstetrics and gynecology and residents in training in this field, titled “Gynecology of the Future—Innovative and New Solutions”, as well as among specialists in obstetrics and gynecology actively working in hospitals. The questionnaire was fully anonymous. The timeframe of the survey was 3 months, from October to December 2023. The respondents were asked to answer six questions. The first question aimed to identify whether they were certified specialists in obstetrics and gynecology or still in training. The remaining questions assessed their knowledge regarding the management of pregnant women after bariatric surgery, including:

- The appropriate interval between bariatric surgery and attempting pregnancy,
- Diagnosis of gestational diabetes in post-bariatric surgery patients,
- Proper dietary recommendations,
- Vitamin and micronutrient supplementation during pregnancy after bariatric surgery,
- Risks associated with such pregnancies.

The survey included both single-choice and multiple-choice questions. The survey’s questions were developed through a consensus process involving experts in the field, including bariatric surgeons, gynecologists, and bariatric dietitians, with the aim of addressing the most significant clinical challenges encountered by these patients. Exclusion criteria included medical specialty of the participants other than obstetrics and gynecology, as well as unclear or incomplete responses, which were excluded from further analysis in this study. Data were collected from 123 certified specialists in obstetrics and gynecology and 37 residents training in obstetrics and gynecology. Results from surveys obtained from five respondents, who declared having had a specialty other than obstetrics and gynecology, were excluded from the study.

The survey used in the study can be found as Appendix A.

### 2.1. Statistical Analysis

Statistical analysis was performed using SPSS Imago Pro 8.0. Cross-tabulations and Pearson’s chi-square test were employed, as the dataset consisted entirely of categorical variables. Statistical significance is assumed when *p* < 0.05.

### 2.2. Ethical Considerations

The study was anonymous and performed in accordance with the ethical standards laid down in the 1964 Declaration of Helsinki and its latter amendments (Fortaleza). Participants were informed about the aim of the study and informed consent was obtained electronically prior to the beginning of the survey. Ethical review and approval were waived for this study because Bioethics Committee approval is not required in Poland for surveys research. We received this statement from the Military Institute of Medicine Ethics Committee on 20 December 2023, code KB/2/23.

## 3. Results

A total of 160 physicians participated in the survey, of whom 123 (76.88%) were specialists in obstetrics and gynecology and 37 (23.13%) were in the process of specializing. In response to question No. 1, “The correct interval that should elapse between bariatric surgery and the commencement of efforts to conceive”, 81 physicians (50.63%) provided a correct answer; the percentage distribution of responses is shown in Table 1. A small proportion of only 48 respondents (30%) provided a right answer to question No. 2, which pertained to the diagnosis of diabetes in pregnancy following bariatric surgery; the percentage distribution of responses is shown in Table 2. In relation to question No. 3, which requested the selection of all correct statements, regarding dietary recommendations for pregnant women who have undergone bariatric surgery, only 8 doctors (5%) provided responses, that were deemed to be correct, but 148 physicians (92.50%) gave at least one correct answer; the percentage distribution of responses is shown in Table 3. The majority of respondents, 86 individuals (53.75%), provided a correct answer to question No. 4, “Vitamin and micronutrient supplementation in pregnancy after bariatric surgery”; the percentage distribution of responses is shown in Table 4. In contrast, only 6 respondents (3.75%) provided an accurate response to question No. 5, which concerned the selection of complications, for which there is an increased risk in patients after bariatric surgery, but 151 physicians (94.38%) gave at least one correct answer; the percentage distribution of responses is shown in Table 5. It is noteworthy that only one physician provided all correct responses, that is 0.625% of our respondents. However, it is important to highlight that in all questions, except for question No. 2, the most frequently chosen answer was the correct answer or, in multiple-choice questions, one of the correct answers. In question No. 2, regarding the diabetes diagnosis in pregnancy after bariatric surgery, the most frequently chosen answer was “Every patient after bariatric surgery should have a 75 g oral glucose load test at 12 Hbd and between 24 and 28 Hbd”, while the correct answer (“Performance of the 75 g oral glucose load test is contraindicated in patients after bariatric surgery and should be replaced by alternative forms of assessing glycemic levels”) took second place—38.75% vs. 30%. On the other hand, the least frequently selected answers were incorrect ones, namely, in question No. 1 “It is not necessary to maintain a time interval” (selected by 3.75% of respondents), in question No. 2 “Not needed, every patient after bariatric surgery should be treated as a patient with gestational diabetes” (selected by 3.13% of respondents), in question No. 3 “The last meal of the day should be eaten by the patient no later than 6:00 p.m.” (selected by 21.88% of respondents), in question No. 4 “Not necessary when following a well-balanced diet” (selected by 1.88% of respondents), and in question No. 5, “High birth weight of the newborn” (selected by 25.63% of respondents). It is worth noting that in question No. 4 (about vitamin and micronutrient supplementation in pregnancy after bariatric surgery), the only respondents who indicated the least frequently chosen answer (“Not necessary when following a well-balanced diet”) as correct were specialists in obstetrics and gynecology.

A correlation was observed between responses to the multiple-choice questions—“There is an increased risk in pregnancy in a patient after bariatric surgery” and “Please select all correct statements about diet in pregnancy after bariatric surgery”. Those who selected the answers “Meals should be eaten in small portions, always sipped with a small amount of liquid”, “Solid foods and beverages should not be combined at one meal” and “The patient should consume high-protein foods because of the increased risk of protein deficiency” were significantly more likely (*p*-value < 0.05) to answer the question about increased risks of complications in pregnancy after bariatric surgery correctly. Another statistically significant correlation was that the majority of respondents, who answered correctly to the question about the diagnosis of diabetes in pregnant women after bariatric surgery, also answered correctly to the question about the correct interval between surgery and pregnancy (*p*-value < 0.05). One more noteworthy correlation should also be highlighted is that all respondents who marked the answer “Not necessary, every patient after bariatric surgery should be treated as a patient with gestational diabetes” when asked about diabetes diagnosis in pregnancy after bariatric surgery, in response to the question regarding potential risks in pregnancy after bariatric surgery consequently identified diabetes in pregnancy as a risk. Due to the limited sample size, it is not possible to determine the statistical significance of this relationship, but it is worth emphasizing. Despite the fact that a significant proportion of respondents provided accurate responses to both the question regarding supplementation and the one concerning the diagnosis of diabetes, no statistically significant correlation was found between the answers to these two questions in this case, indicating that, based on the data collected, there is no clear relationship between these two aspects of knowledge among the physicians. Upon conducting a thorough analysis of the remaining questions and the responses provided, we were unable to identify any additional statistically significant correlations or noteworthy trends that could suggest clear associations between the answers given by the participants.

## 4. Discussion

Given that the Polish Society of Gynecologists and Obstetricians (PTGiP) guidelines on managing pregnancy after bariatric surgery are limited, we aimed to assess the knowledge of gynecologists on this topic. Despite the fact that the majority of survey respondents (74.50%) were specialists in obstetrics and gynecology, their knowledge and understanding of managing such patients appeared to be insufficient.

One of the objectives of this analysis was to assess whether there was a statistically significant difference in the percentage of correct answers between two groups of respondents: those who were already specialists in gynecology and obstetrics and those who were still in the process of specializing in this field. The responses provided by specialists to questions related to supplementation, the appropriate time interval between bariatric surgery and the commencement of pregnancy, and dietary recommendations for pregnant women following bariatric surgery were generally more accurate. On the other hand, residents demonstrated slightly better knowledge when answering questions regarding the diagnosis of diabetes during pregnancy following bariatric surgery, as well as identifying the increased risk of complications in pregnancy for patients who had undergone such surgeries. Despite these observed differences in the responses between the two groups, it is important to note that the differences were minimal, and no statistically significant correlation or substantial variation were found between the two groups’ answers. Therefore, we are unable to draw any definitive conclusions about which group demonstrated superior competence in addressing the questions posed. Furthermore, it is crucial to consider the significant imbalance in the number of specialists compared to residents, as this imbalance may have influenced the results and may contribute to the minimal differences observed between the two groups in this study.

### 4.1. Optimum Interval Between BS and Time of Conception

One of the key aspects of managing women post-bariatric surgery (BS) is the recommended interval between surgery and conception. According to the ACOG guidelines (The American College of Obstetricians and Gynecologists, 2021), this interval should be 1–2 years [9]. This recommendation is based on the period of rapid weight loss and potential nutritional deficiencies, which could adversely affect fetal development [10,11,12]. In our survey, only 50.3% of respondents identified the correct interval, highlighting significant gaps in knowledge at the outset.

### 4.2. Diagnosing Gestational Diabetes in Women After BS

Bariatric surgery affects carbohydrate absorption, complicating the diagnosis of gestational diabetes. The standard OGTT (oral glucose tolerance test) is unreliable due to rapid gastric emptying (dumping syndrome) and variable glycemic responses [13,14]. Nevertheless, nearly 40% of respondents believed that OGTT should be performed in all post-BS patients, and about 30% assumed that gestational diabetes diagnosis in post-BS pregnancies does not differ from standard approaches. It is essential to develop personalized diagnostic plans tailored to the individual needs of these patients—an aspect recognized by only 30% of respondents.

### 4.3. Diet in Pregnancy After BS

Diet is a critical aspect of managing pregnant women after BS. Nutritional needs in this group are influenced by both the demands of pregnancy and the consequences of the surgery. Proper meal planning can prevent nutrient deficiencies and reduce health risks for both the mother and fetus [15]. However, our survey revealed limited awareness among specialists. While 51% believed the recommended calorie intake should be 1500–2000 kcal/day, this amount is inadequate and may lead to maternal and fetal deficiencies [6,14]. Additionally, 21% incorrectly stated that the last meal of the day should be eaten by 6:00 p.m., whereas it should ideally be consumed 2–3 h before bedtime to mitigate reflux—a common issue post-BS. Meals should also be small due to reduced gastric capacity, and patients should avoid drinking fluids with meals to prevent a sensation of fullness and impaired digestion. Alarmingly, 66% of respondents answered this question incorrectly.

Furthermore, when faced with sudden hunger, a patient should avoid consuming small, high-carbohydrate snacks [13], as these can cause glucose fluctuations—a point misunderstood by 37% of respondents. Encouragingly, 53% recognized the importance of high-protein foods [12], and 87% acknowledged the need for regular consultations with an experienced dietitian [16].

### 4.4. Vitamin and Micronutrient Supplementation in Pregnancy After BS

Vitamin and micronutrient deficiencies pose one of the major challenges in pregnancy after BS. Women in this group are at higher risk of deficiencies in iron, vitamin B12, folic acid, calcium, and vitamin D—key elements for maternal health and fetal development [8,17,18,19]. In our survey, 54% of respondents understood the importance of prenatal multivitamin supplementation, as well as additional supplementation of iron, calcium, vitamin D, and vitamin B12. It is recommended to begin supplementation before conception and to continue throughout pregnancy and breastfeeding.

### 4.5. Risks Associated with Pregnancy After BS

Our findings indicate that 62.4% of respondents identified low birth weight as a condition with increased risk for post-BS pregnancies. Maternal weight loss can lead to nutrient deficiencies, affecting fetal growth [8]. The literature emphasizes the need to monitor vitamin and micronutrient levels, such as iron and vitamin B12, to mitigate this risk [20,21,22].

Similarly, 61.2% recognized intrauterine growth restriction as a significant concern. Malabsorption following BS can limit the availability of critical nutrients for fetal growth [5,16,23,24,25].

Gestational diabetes was highlighted by 67.9% as a major risk. Despite weight reduction, metabolic disturbances such as insulin resistance may persist, necessitating close glycemic monitoring.

Preeclampsia was noted by 47.9% of respondents. While BS reduces the overall risk of hypertension, nutritional deficiencies and metabolic changes can still contribute to this complication during pregnancy [23].

Anemia, indicated by 76.4% of respondents, emerged as one of the most common complications in post-BS pregnancies. Impaired absorption of iron, folic acid, and vitamin B12 increases the risk of anemia for both the mother and fetus, necessitating supplementation [22].

Furthermore, 55.2% acknowledged the elevated likelihood of cesarean delivery. Although BS decreases risks associated with obesity-related complications, other factors, such as weakened abdominal muscles or maternal health concerns, may necessitate surgical delivery [25].

Finally, the underperformance of gynecologists can be attributed to numerous factors, including deficiencies in specialist training and the poorly structured post-specialist training opportunities available in Poland. Additionally, the limited number of patients seen by gynecologists following BS could be a contributing factor to the observed lack of knowledge. The available guidelines are underdeveloped and not sufficiently widespread, omitting numerous clinically significant aspects. In this case, potential solutions may include the incorporation of this subject matter into the training of gynecologists and the enhancement of post-specialization training opportunities in Poland. Furthermore, it is imperative to enhance the development of guidelines for the treatment of such patients and to disseminate them more extensively.

### 4.6. Limitations of the Study

Several limitations should be considered when interpreting the results, despite the valuable insights provided by our study:

Potential sampling bias—the process of recruiting respondents may have resulted in an overrepresentation of physicians already interested in the topic of bariatric surgery, which may have affected the results of the survey.

Homogenous test sample—all of our respondents were physicians practicing in Poland, which made it impossible for us to assess how knowledge of the management of pregnant women after bariatric surgery is evolving in other regions.

Resident and specialist group size differences—it is imperative to acknowledge the substantial discrepancy in the number of specialists (n = 123) and residents (n = 37). This disparity may have influenced the outcomes and contributed to the negligible differences observed between the two groups in this investigation.

Lack of similar studies—the lack of comparable studies on the knowledge of gynecologists regarding the management of pregnant women post-bariatric surgery severely limits the ability to compare results from Poland with those from other regions. This limitation also affects the overall interpretation and drawing of conclusions, and consequently, the identification of solutions to address the deficiencies in the knowledge of gynecologists regarding the management of such patients.

## 5. Conclusions

The level of knowledge among Polish obstetricians-gynecologists regarding the care of pregnant women after bariatric surgery is currently insufficient and requires urgent improvement. Understanding the specifics of managing pregnancies in patients who have undergone bariatric surgery, including potential complications and unique needs, is essential to ensuring the safety and health of both the mother and the child. Given the global rise in obesity and the associated increase in bariatric surgeries, it is anticipated that more women of reproductive age will undergo such procedures.

To address the challenges posed by this situation, it is necessary to implement additional training and educational programs for obstetricians-gynecologists and not rely solely on the knowledge gained from specialty training, as well as modifications to the curricula for medical students specializing in gynecology. Such initiatives will enable physicians and medical trainees to better understand the specific needs of this patient group, improve the quality of care, and minimize the risk of complications. In addition, it is imperative to ensure that the guidelines for these patients are both expanded upon and propagated. These measures are critical to adapting healthcare to the changing demographic landscape and enhancing the health and safety of women after bariatric surgery.

## Figures and Tables

**Table 1:** Answers distribution in Question 1.

The Correct Interval That Should Elapse Between Bariatric Surgery and the Commencement of Efforts to Conceive		
Answers	Respondents	Percent
6 months	63	39.38%
1–2 years	81	50.63%
more than 2.5 years	10	6.25%
It is not necessary to maintain a time interval	6	3.75%

**Table 2:** Answers distribution in Question 2.

Diabetes Diagnosis in Pregnancy After Bariatric Surgery:		
Answers	Respondents	Percent
Not needed, every patient after bariatric surgery should be treated as a patient with gestational diabetes	5	3.13%
Not different from the standard diagnosis in pregnancy, the patient should have an oral 75 g glucose load test between 24 and 28 Hbd	45	28.13%
Every patient after bariatric surgery should have a 75 g oral glucose load test at 12 Hbd and between 24 and 28 Hbd	62	38.75%
Performance of the 75 g oral glucose load test is contraindicated in patients after bariatric surgery and should be replaced by alternative forms of assessing glycemic levels	48	30.00%

**Table 3:** Answers distribution in Question 3.

Please Select All Correct Statements About Diet in Pregnancy After Bariatric Surgery:		
Answers	Respondents	Percent
The recommended calorie diet during pregnancy is between 1500–2000 kcal per day, so as to maintain a stable rate of weight loss	83	51.88%
The last meal of the day should be eaten by the patient no later than 6:00 p.m.	35	21.88%
Meals should be eaten in small portions, always sipped with a small amount of liquid	107	66.88%
Solid foods and drinks should not be combined at one meal	64	40.00%
In a situation of sudden hunger, it is recommended to consume a small meal high in short-acting carbohydrates to quickly compensate for hypoglycemia	59	36.88%
Patient should consume high-protein foods due to increased risk of protein deficiency	88	55.00%
Regular consultation with a dietician experienced in caring for patients after bariatric surgery is recommended	139	86.88%

**Table 4:** Answers distribution in Question 4.

Vitamin and Micronutrient Supplementation in Pregnancy After Bariatric Surgery:		
Answers	Respondents	Percent
Not necessary when following a well-balanced diet	3	1.88%
Sufficient supplementation is provided by taking a multivitamin formula for pregnant women	6	3.75%
The patient should always take a multivitamin preparation for pregnant women and additional supplementation of iron, calcium, vitamin D and B12 preparation	86	53.75%
The patient should take a multivitamin preparation for pregnant women and additional supplementation with a preparation of iron, calcium, vitamin D and B12 only in case of confirmed deficiencies	62	38.75%

**Table 5:** Answers distribution in Question 5.

There Is an Increased Risk in Pregnancy in a Patient After Bariatric Surgery (Multiple Choice Question)		
Answers	Respondents	Percent
High birth weight of the newborn	83	51.88%
Low birth weight of the newborn	35	21.88%
Stunting of intrauterine growth in the fetus	107	66.88%
Diabetes in pregnancy	64	40.00%
Pre-eclampsia	59	36.88%
Anemia	88	55.00%
Termination of pregnancy by caesarean section	139	86.88%

## Data Availability

The data will be available from the corresponding author on request.

## Abbreviations

The following abbreviations are used in this manuscript:

WHO	World Health Organization
BMI	Body Mass Index
BS	Bariatric Surgery
OGTT	Oral Glucose Tolerance Test
GDM	Gestational Diabetes Mellitus
FGR	Fetal Growth Restriction
IFSO	International Federation for the Surgery of Obesity and Metabolic Disorders
PTGiP	Polish Society of Gynecologists and Obstetricians
ACOG	The American College of Obstetricians and Gynecologists

## Appendix A. A Study on Recommendations for Pregnancy Management in Patients After Metabolic Bariatric Surgery

Dear Sir/Madam,

Thank you for your willingness to participate in our study. The questionnaire includes 6 questions. Your answers will help optimize the level of care for pregnant patients after metabolic bariatric surgeries. Completing the survey below will take you about 5 min. We ask you to answer truthfully. Your answers will be completely anonymous and analyzed only by the team of researchers. Filling the survey is equivalent with acceptance of participation in study.

Yours faithfully,
Team of researchers

1. What is Mr./Mrs. specialty?
  - I am in the process of specializing in obstetrics and gynecology
  - Specialist in obstetrics and gynecology
  - Other specialty
2. The correct time interval that should elapse between bariatric surgery and conception is:
  - 6 months
  - 1–2 years
  - Above 2.5 years
  - There is no need to observe the time interval, the patient should start trying to get pregnant as soon as possible after the surgery

Correct answer: b

3. Diagnosing gestational diabetes mellitus after bariatric surgery:
  - It is not necessary, each post—bariatric patient should be considered as a patient with diabetes during pregnancy
  - It does not differ from standard diagnostics in pregnancy, the patient should have a 75 g oral glucose tolerance test between 24 and 28 Hbd
  - Each patient after bariatric surgery should have a 75 g oral glucose tolerance test on 12 Hbd and between 24 and 28 Hbd
  - Having a 75 g oral glucose tolerance test is contraindicated in patients after bariatric surgery and should be replaced with alternative forms of assessing glycemia levels

Correct answer: d

4. Please select all correct statements about diet during pregnancy after bariatric surgery:
  - The recommended caloric content of the diet during pregnancy is between 1500–2000 kcal per day to maintain a stable rate of weight loss
  - The patient should eat the last meal of the day no later than 6 p.m.
  - Meals should be eaten in small portions, always followed by a small amount of fluid
  - Solid foods and drinks should not be combined in one meal
  - In the case of a sudden feeling of hunger, it is recommended to eat a small meal rich in short-acting carbohydrates to quickly compensate hypoglycemia
  - The patient should eat high-protein foods due to the increased risk of protein deficiency
  - Dietitian care is recommended regularly throughout the pregnancy, performed by a bariatric dietitian

Correct answers: d, f, g

5. Supplementation of vitamins and microelements during pregnancy after bariatric surgery:
  - It is not necessary in case of the following a properly balanced diet
  - Sufficient supplementation is ensured by taking a multivitamin for pregnant women
  - The patient should take a multivitamin for pregnant women and, as a standard, additional supplementation with iron, calcium, vitamin D and B12
  - The patient should take a multivitamin preparation for pregnant women and additional supplementation with iron, calcium, vitamin D and B12 only in case of confirmed deficiencies

Correct answer: c

6. During pregnancy, a patient after bariatric surgery has an increased risk of (please select all correct statements):
  - High birth weight of the newborn
  - Low birth weight of the newborn
  - Intrauterine growth restriction (IUGR)
  - Diabetes during pregnancy
  - Preeclampsia (PE)
  - Anemia
  - Termination of pregnancy by cesarean section

Correct answers: b, c, f, g
